# Psychological resilience correlates with EEG source-space brain network flexibility

**DOI:** 10.1162/netn_a_00079

**Published:** 2019-03-01

**Authors:** Véronique Paban, Julien Modolo, Ahmad Mheich, Mahmoud Hassan

**Affiliations:** Aix Marseille University, CNRS, LNSC, Marseille, France; University of Rennes, INSERM, LTSI-U1099, F-35000 Rennes, France; University of Rennes, INSERM, LTSI-U1099, F-35000 Rennes, France; University of Rennes, INSERM, LTSI-U1099, F-35000 Rennes, France

**Keywords:** EEG source connectivity, Psychological resilience, Resting state, Flexibility

## Abstract

We aimed at identifying the potential relationship between the dynamical properties of the human functional network at rest and one of the most prominent traits of personality, namely resilience. To tackle this issue, we used resting-state EEG data recorded from 45 healthy subjects. Resilience was quantified using the 10-item Connor-Davidson Resilience Scale (CD-RISC). By using a sliding windows approach, brain networks in each EEG frequency band (delta, theta, alpha, and beta) were constructed using the EEG source-space connectivity method. Brain networks dynamics were evaluated using the network flexibility, linked with the tendency of a given node to change its modular affiliation over time. The results revealed a negative correlation between the psychological resilience and the brain network flexibility for a limited number of brain regions within the delta, alpha, and beta bands. This study provides evidence that network flexibility, a metric of dynamic functional networks, is strongly correlated with psychological resilience as assessed from personality testing. Beyond this proof-of-principle that reliable EEG-based quantities representative of personality traits can be identified, this motivates further investigation regarding the full spectrum of personality aspects and their relationship with functional networks.

## Author Summary

In this study, we investigated the possible correlation between one of the most important personality traits, *resilience*, with a metric of dynamic functional networks called *flexibility*. From EEG resting-state recordings in *N* = 45 volunteers, we unveiled such a correlation and identified the brain regions involved in psychological resilience, from frequency-specific networks.

## INTRODUCTION

An evolving field of neuroscience aims to reveal the neural substrates of personality, referring to the relatively stable character of an individual that influences her or his long-term behavioral style (Dubois et al., 2018). A key personality character is *resilience*, defined as the ability to adapt to stress, adversity, and negative events and cope actively with life challenges (Fletcher & Sarkar, 2013; Luthar, 2003; Rutter, 2006). Recently, a multisystem model of resilience has been proposed by Liu et al. (2017), in which resilience comprises three structures: (1) the innermost layer, which comprises the physiological, biological, and demographic profiles of an individual; (2) the intermediate layer, which includes internal factors such as family, friends, and personal experiences; and (3) the outermost layer, which corresponds to external resilience such as access to healthcare and social services. However, except a few efforts (Kong et al., 2015; Reynaud et al., 2013; Waugh & Koster, 2015), the neuralsubtracts of this complex personality trait remain unclear.

The last decade has witnessed an increase of studies that consider the human brain as a large-scale network. It is thus unsurprising that network neuroscience, which uses tools from graph theory to better understand neural systems (Bassett & Sporns, 2017), has become one of the most promising approaches to link behavior to brain function, including personality traits (Markett et al., 2018). In the network neuroscience model, the human brain is summarized by a set of nodes representing brain regions and a set of edges representing the connections between these brain regions. The magneto/electro-encephalography (M/EEG) source-space networks provide a unique direct and noninvasive access to those electrophysiological brain networks, at the milliseconds temporal scale (Hassan & Wendling, 2018; Mheich et al., 2018). The excellent time precision of this method allows the tracking of the brain networks dynamics at an unprecedented timescale. In this paper, we aimed to test the hypothesis that metrics derived from brain networks dynamics can be correlated to resilience scores.

To test this hypothesis, we used resting-state EEG data recorded from *N* = 45 healthy subjects. Resilience was quantified using the Connor-Davidson Resilience Scale (CD-RISC) (Campbell-Sills & Stein, 2007), where higher scores correspond to greater resilience. Brain networks in each frequency band (delta, theta, alpha, and beta) were constructed using the EEG source-space connectivity method. By applying multislice modularity algorithms on the dynamic networks, the reconfiguration of EEG source-space networks was quantified using flexibility, defined as how often a given node changes its modular affiliation over time, computed at the level of each brain region. The choice of flexibility as an appropriate metric was made following a recent study discussing the relationship between functional networks and personality traits (Tompson et al., 2018). Indeed, flexibility captures, in an intuitive manner, the dynamics of functional networks. Results revealed essentially a negative correlation between psychological resilience and EEG-based brain functional network flexibility.

## RESULTS

**Figure 1.**
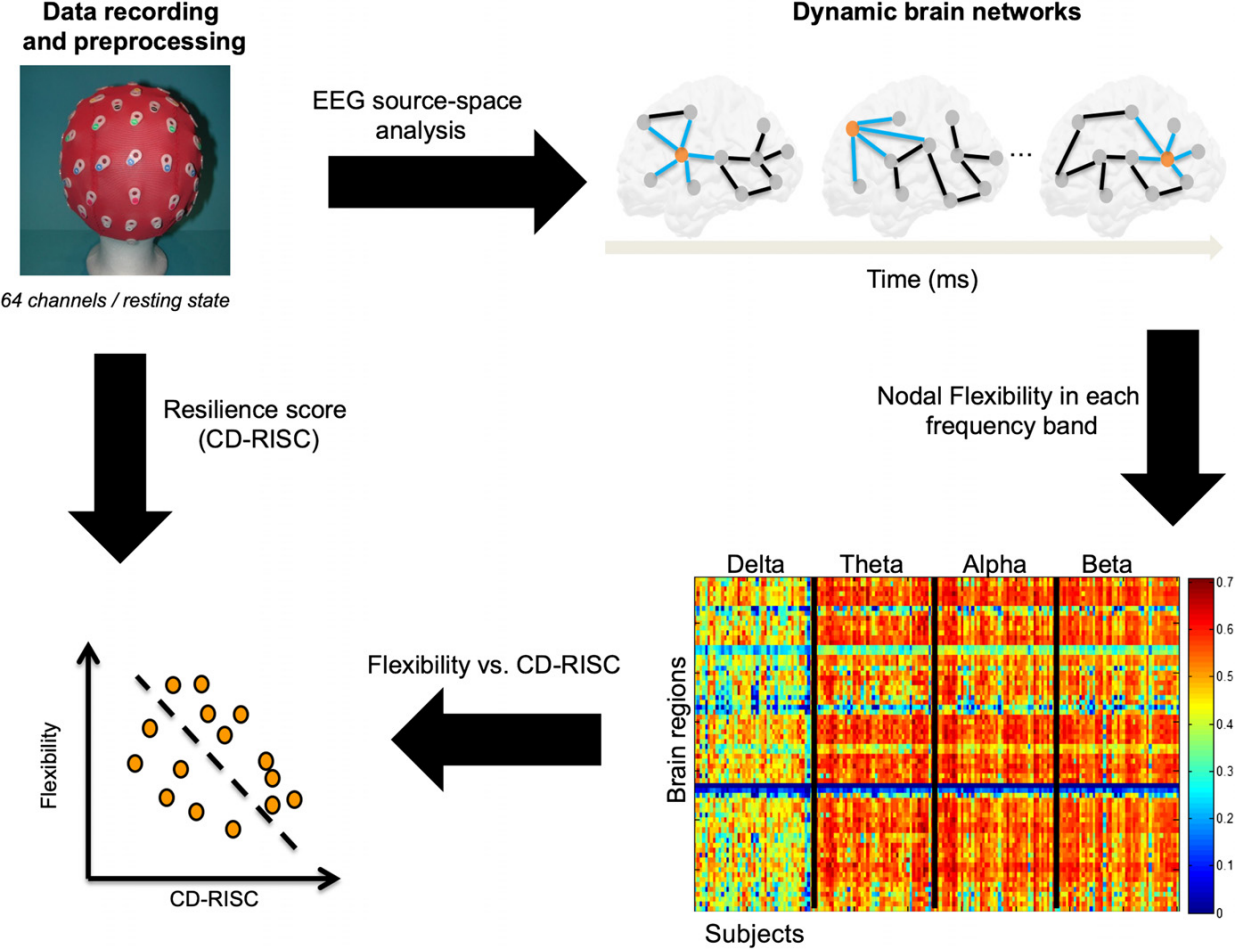
Experimental protocol and data analysis pipeline.

The study is summarized in Figure 1. First, EEG data were recorded and preprocessed. Second, the dynamic networks were estimated using the EEG source-space approach, giving a set of brain network at the given time period for each frequency band. Third, the flexibility of each brain region in each frequency band were computed for each subject. Finally, the correlation between the brain regions flexibility and the resilience score (CD-RISC) was computed.

**Figure 2.**
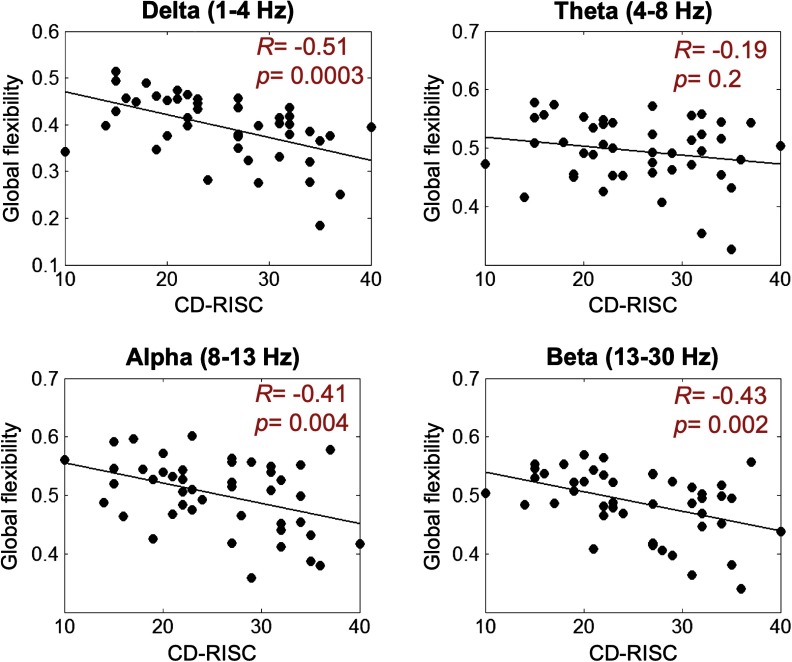
Correlation between the global flexibility (averaged over all brain regions) and the CD-RISC score.

Then, we computed the Pearson correlation between the resilience score and the global flexibility (averaged over all brain regions for each subject in each frequency band). The corresponding results are presented in Figure 2. For all frequency bands, a negative correlation was observed. This negative correlation was significant for delta (*R* = −0.51, *p* = 0.0003), alpha (*R* = −0.41, *p* = 0.004), and beta (*R* = −0.43, *p* = 0.002) bands, but nonsignificant for the theta band (*R* = −0.19, *p* = 0.2).

**Figure 3.**
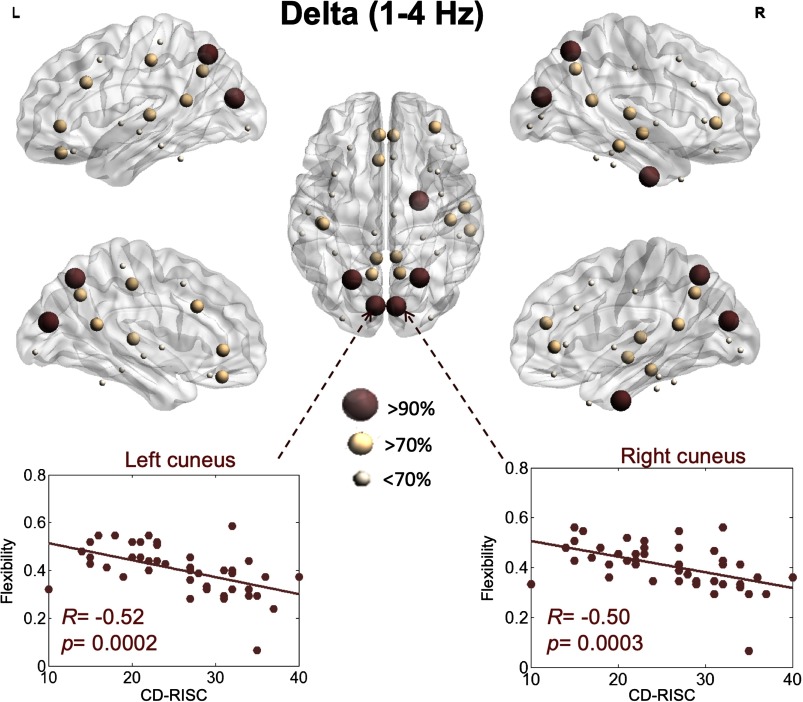
Illustration of the brain regions for which flexibility is significantly correlated with resilience within the delta band.

We then focused on the correlation between flexibility and the resilience score at the level of each brain region. In the delta band, brain regions that were significant and correlated with the CD-SCORE are illustrated in Figure 3. The flexibility of the left cuneus (*R* = −0.52, *p* = 0.0002, false discovery rate [FDR] corrected), the right cuneus (*R* = −0.50, *p* = 0.0004, FDR corrected), the left superior parietal (*R* = −0.49, *p* = 0.0005, FDR corrected), the right superior parietal (*R* = −0.49, *p* = 0.0006, FDR corrected), and the right entorhinal (*R* = −0.45, *p* = 0.0006, FDR corrected) had the highest (>90%) correlation with the resilience score (e.g., the brain regions that had the highest 90% correlation between the resilience score and the network-based score).

**Figure 4.**
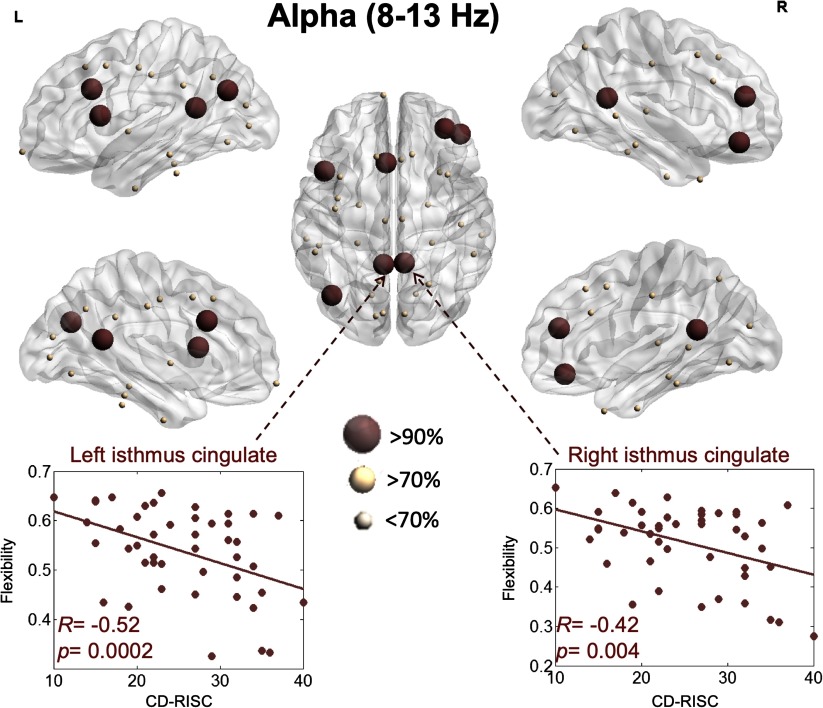
Illustration of the brain regions for which flexibility is significantly correlated with resilience within the alpha band.

The results for the alpha band are presented in Figure 4. The flexibility of the left caudal anterior cingulate (*R* = −0.46, *p* = 0.001, FDR corrected), the right rostral middle frontal (*R* = −0.46, *p* = 0.001, FDR corrected), the right pars orbitalis (*R* = −0.45, *p* = 0.001, FDR corrected), the left inferior parietal (*R* = −0.44, *p* = 0.002, FDR corrected), the left isthmus cingulate (*R* = −0.43, *p* = 0.003, FDR corrected), the right isthmus cingulate (*R* = −0.42, *p* = 0.004, FDR corrected), and the right pars opercularis (*R* = −0.41, *p* = 0.004, FDR corrected) showed the highest correlations with the resilience score.

**Figure 5.**
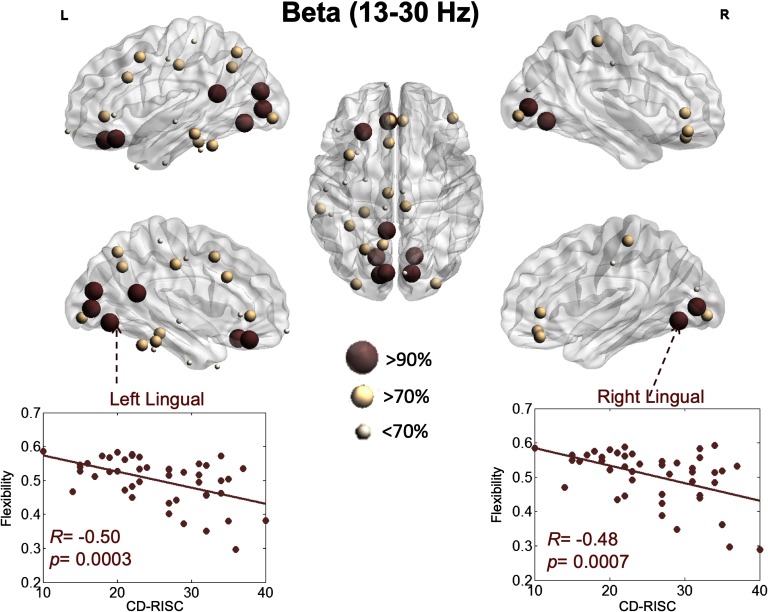
Illustration of the brain regions for which flexibility is significantly correlated with resilience within the beta band.

Results regarding the beta band revealed a more pronounced implication of visual networks (Figure 5). Specifically, the flexibility of the left lingual (*R* = −0.50, *p* = 0.0004, FDR corrected), right lingual (*R* = −0.48, *p* = 0.0007, FDR corrected), left pericalcarine (*R* = −0.48, *p* = 0.0007, FDR corrected), right pericalcarine (*R* = −0.47, *p* = 0.0009, FDR corrected), left cuneus (*R* = −0.46, *p* = 0.001, FDR corrected), left isthmus cingulate (*R* = −0.46, *p* = 0.001, FDR corrected), left medial orbitofrontal (*R* = −0.45, *p* = 0.001, FDR corrected), and left lateral orbitofrontal (*R* = −0.45, *p* = 0.001, FDR corrected) had the highest correlation with the resilience score.

## DISCUSSION

Our results reveal a robust and direct relationship between the network flexibility (as quantified through graph theory) of human functional brain networks and resilience (as measured with neuropsychological testing). This result has been obtained using noninvasive brain recordings (EEG), in the absence of any specific task or stimulus (resting-state recordings), for a relatively large sample size (*N* = 45). Specifically, the network flexibility of specific brain regions is shown to be significantly decreased with a higher resilience score, which can appear nonintuitive: if resilience refers to a capacity to cope with external stress and adapt, it could imply that brain networks can reconfigure to adapt to these changes. Our results point at the opposite: most brain regions negatively correlated with the resilience score are part of the “rich club” (“core” functional network of brain regions [Van Den Heuvel & Sporns, 2011]) and illustrate that the brain core network is less flexible in resilient subjects. This suggests that a stable core network would allow preventing excessive reconfiguration of functional brain networks following external stress factors, for example. From a fundamental perspective, these results also suggest that there exists a brain network of critical importance, which underlies one of the most prominent personality traits (resilience). This raises the possibility to probe further potential brain network correlates of other personality traits in the future. Furthermore, this link between core network structures and resilience is fundamental, since it can be detected noninvasively even in resting-state recordings. Another insight from our analysis is that dynamic but not static network analysis was able to reveal this association between resilience and a graph theory metric. Static analyses were realized using clustering coefficient and participation coefficient, and did not reveal any significant correlation with the resilience score (see Supporting Information Figure S1 and Figure S2; Paban, Modolo, Mheich, & Hassan, 2019). Therefore, this highlights the importance of the dynamic reconfiguration of brain networks in shaping behavior. Recent studies have emphasized the interest to characterize the architecture underlying these dynamics, for example, by identifying the “life duration” and transition rates of these frequency-specific networks (Vidaurre et al., 2018). Overall, as emphasized in a recent perspective paper by Tompson et al. (2018), there is a significant potential regarding the use of network neuroscience tools to unveil the brain networks underlying personality traits, with flexibility and integration being especially promising.

### Neural Substrates of Resilience

Functional neuroimaging studies examining brain networks associated with resilience are limited. Most of them mainly focused on patient populations, such as depressive, traumatized, and posttraumatic stress disorders, in which alterations where described in regions of the brain involved in emotion and stress regulation circuitry (see van der Werff et al., 2013, for review). In healthy adults, to our knowledge, only one resting-state fMRI (rs-fMRI) experiment has been reported to date. Interestingly, Kong et al. (2015) reported that psychological resilience had significant negative correlations with the rs-fMRI signals, but in regions such as the bilateral insula, right dorsal, and rostral anterior cingulate cortex. Our study did not highlight the same brain regions. Our dynamic network analysis identified regions belonging to the “core” functional network described by Van Den Heuvel & Sporns (2011). Those regions are supposed to play a central role in establishing and maintaining efficient global brain communication. They have been shown to be involved in cognitive processes related to top-down attentional control (superior parietal cortex [Sestieri et al., 2017]), decision-making (subdivision of the orbitofrontal cortex [Schuck et al., 2018]), cognitive regulation of behavior (caudal anterior cingulate [Bush et al., 2000]), and spatial location (entorhinal cortex [Kim & Maguire, 2018]). Our data also highlighted the regions participating in the visual network (e.g., cuneus, the lingual gyrus, and the pericalcarine cortex). This network oscillated in fast frequency band (beta), which is thought to reflect different aspects of sensory information processing (Hong et al., 2008). Another region negatively correlated with the resilience score comprised the inferior parietal cortex. It has been shown to be implicated in major cognitive functions, including visuo-spatial attention and visual memory (Egner et al., 2008).

The present study provided evidence of neuronal substrates of resilience. In healthy adults, it seems to make sense that the ability of individuals to cope actively with life’s challenges (Fletcher & Sarkar, 2013) requests brain regions involved in high-level cognitive processes. To be efficient, these regions should have a modular organization stable over time. As suggested by Betzel et al. (2016), such network architecture limits information exchange across modules, which allows a specialized information processing. Such modular organization seemed to be an important factor in maintaining a stable equilibrium of psychological and physical functioning when facing adversity, ranging from daily problems to major life events (Bonanno, 2004). When this is not the case, that is, when few brain regions showed high modular affiliation exchanges, one may assume that they multiply their contributions and so participate in a multitude of brain functioning. In this context, subjects had low-resilient scores, meaning that they exhibit passive coping strategies, high emotional load, ruminative and depressogenic thinking, and low life satisfaction (Davydov et al., 2010).

### Methodological Considerations

First, in the present study we used a template source-space, instead of a subject-specific one, that is, the same structural MRIs of healthy subjects were used in our EEG functional connectivity analysis. In the case of healthy subjects, it was reported that coregistration with the template yielded largely consistent connectivity and network estimates compared with native MRI (Douw et al., 2018). However, in the case of patient-related studies, it is more preferred to use patient-specific MRIs.

Second, it is important to keep in mind that computing the functional connectivity at the scalp level is severely corrupted by the volume conduction problem (Brookes et al., 2014; Lai et al., 2018; Schoffelen & Gross, 2009). M/EEG connectivity analysis at the source-space was shown to reduce this effect as connectivity is applied to “local” time series generated by cortical neuronal assemblies modeled, for instance, as current dipole sources. Yet, the “mixing effects” can also occur in the source-space. To address this issue, a number of methods were developed based mostly on the rejection of zero-lag correlation. In particular, “unmixing” methods, called “leakage correction” (such as the orthogonalization approach [Colclough et al., 2015]), have been reported, which force the reconstructed signals to have zero cross-correlation at lag zero. Here we preferred to use the phase locking value (PLV) metric and keep these zero-lag correlations, as several experimental studies reported the importance (in many conditions) of the zero-lag synchronizations in the human brain. Nevertheless, we believe that there is no ideal solution yet for this methdological issue and that further efforts are needed to completely solve the spatial leakage problem.

Third, we have performed the whole analysis in all EEG frequency bands in order to avoid making any a priori judgments about the involved rhythms. The reported effects were the same when other thresholds were used. However, it should be kept in mind that standard EEG frequency bands are increasingly associated with specific functional roles in brain-scale information processing. As an exemple, alpha activity appears to mediate the so-called “gating by inhibition” mechanism involved in information routing (Bonnefond et al., 2017) through specific nodes of the cortical network while inhibiting irrelevant regions. On the other hand, theta oscillations appear to be involved in locking distant brain regions to enable further processing (Lisman & Jensen, 2013). Since different frequency bands appear linked with specific functional roles, there is no reason a priori that psychological resilience results from an interplay between all of those frequency bands. Therefore, we believe that our results point at those oscillations that are functionally linked with resilience, and that theta oscillations do not appear to have a specific role in this case.

Finally, although EEG source connectivity has improved the spatial resolution of EEG, the networks identified are, however, still limited to cortical gray matter. In fact, the localization of subcortical structures remains extremely challenging using EEG technology, namely because of anatomical and analytical reasons. For example, unlike the layered cortex, a subcortical region does not have a sufficient organization of pyramidal cells to give rise to localizable scalp-recorded EEG.

## MATERIAL AND METHODS

### Participants

A total of 45 healthy subjects were recruited (22 women). The mean age was 34.7 years old (*SD* = 9.1 years, range = 18–55). Education ranged from 10 years of schooling to a PhD degree. None of the volunteers reported taking any medication or drugs, nor suffering from any past or present neurological or psychiatric disease. After receiving approval from the Aix-Marseille university ethics committee according to the Declaration of Helsinki, participants filled out the CD-RISC questionnaire at home approximately 1 week before the EEG experiment. Written informed consent was obtained from all participants prior to study onset.

### Measuring Psychological Resilience

The Connor-Davidson Resilience Scale (CD-RISC; Connor & Davidson, 2003) is a 25-item scale that measures the ability to cope with adversity. A 10-items version (CD-RISC 10) of this scale has been developed by Campbell-Sills and Stein (Campbell-Sills & Stein, 2007). A 10-item version validated for French speaking populations was used in the present study (Guihard et al., 2018; Scali et al., 2012). The 10 items are rated on a five-point Likert scale that ranges from 0 (not at all) to 4 (true nearly all of the time). Higher scores correspond to greater resilience. This scale demonstrated good internal consistency and construct validity (Campbell-Sills & Stein, 2007). In our sample, the CD-RISC 10 exhibited a reliability of α = 0.90.

### Data Acquisition and Preprocessing

Each EEG session consisted of a 10-min resting period with the participant’s eyes closed (Paban et al., 2018). Participants were seated in a dimly lit room, were instructed to close their eyes, and then to simply relax until they were informed that they could open their eyes. Participants were instructed that the resting period would last approximately 10 min. The eyes-closed resting EEG recordings protocol was chosen to minimize movement and sensory input effects on electrical brain activity. EEG data were collected using a 64-channel Biosemi ActiveTwo system (Biosemi Instruments, Amsterdam, The Netherlands) positioned according to the standard 10–20 system montage, one electrocardiogram, and two bilateral electro-oculogram electrodes (EOG) for horizontal movements. Nasion-inion and preauricular anatomical measurements were made to locate each individual’s vertex site. Electrode impedances were kept below 20 kOhm. EEG signals are frequently contaminated by several sources of artifacts, which were addressed using the same preprocessing steps as described in several previous studies dealing with EEG resting-state data (Kabbara et al., 2018, 2017; Rizkallah et al., 2018). Briefly, bad channels (signals that are either completely flat or contaminated by movement artifacts) were first identified by visual inspection, complemented by the power spectral density. These bad channels were then recovered using an interpolation procedure implemented in Brainstorm (Tadel et al., 2011) by using neighboring electrodes within a 5-cm radius. Epochs with voltage fluctuations more than +80 μV and less than −80 μV were removed. Five artifact-free epochs of 40-s lengths were selected for each participant. This epoch length was used in a previous study, and was considered as a good compromise between the needed temporal resolution and the results reproducibility (Kabbara et al., 2017). By using a sliding windows approach to compute the functional connectivity, a large number of networks (depend on the analyzed frequency band) were obtained for each 40-s epoch.

### Brain Networks Construction

First, brain networks were reconstructed using the “EEG source-space connectivity” method (Hassan et al., 2014; Hassan & Wendling, 2018), which includes two main steps: (1) reconstruct the dynamics of the cortical sources by solving the inverse problem, and (2) measure the statistical couplings (functional connectivity) between the reconstructed time series. In summary, EEG source connectivity links the recorded EEG signals with the functional relationship between anatomical brain regions (e.g., networks), through the EEG inverse problem that provides the localization of the cortical sources originating these EEG signals. Let us mention that numerous methods exist for both of these two steps (EEG inverse problem and functional connectivity measures). EEGs and MRI template (ICBM152) were coregistered through the identification of anatomical landmarks by using Brainstorm (Tadel et al., 2011). A Desikan-Killiany atlas-based segmentation approach was used, consisting of 68 cortical regions (Desikan et al., 2006). The OpenMEEG (Gramfort et al., 2010) software was used to compute the head model. Here, we used the weighted minimum norm estimate (wMNE) algorithm as an inverse solution. The reconstructed regional time series were filtered in different frequency bands (delta, 1–4 Hz; theta, 4–8 Hz; alpha, 8–13 Hz; and beta, 13–30 Hz). For each frequency band, functional connectivity was computed between the regional time series using the PLV measure (Lachaux et al., 1999). This combination wMNE/PLV was chosen according to a recent model-based comparative study of different inverse/connectivity combinations (Hassan et al., 2017)

Using PLV, dynamic functional connectivity matrices were computed for each epoch using a sliding windows technique. It consists of moving a time window of certain duration δ, and PLV is calculated within each window. As recommended in Lachaux et al. (2000), we selected the smallest window length equal to $\frac{\text{6}}{\text{central}\;\text{frequency}}$, where 6 is the number of “cycles” at the given frequency band. For instance, in the theta band, since the central frequency (Cf) equals to 6 Hz, δ equals 1 s, and δ = 279 ms (Cf = 21.5 Hz) in beta band. Thus, for each epoch, 33 networks were obtained for the theta band, and 130 networks in the beta band. The same calculation was adopted for other frequency bands. Finally, we kept only the strongest 10% of connections.

### Network Modularity and Flexibility

The obtained dynamic matrices were divided into time-dependent modules by using the multislice community detection approach described in Mucha et al. (2010). It consists of introducing a parameter that associates nodes across time, before applying the modularity procedure, and is defined as (one modularity value is computed for each frequency band of interest; Sporns & Betzel, 2016):

$$Q\left(\gamma ,\omega \right)=\frac{1}{2\mu }\underset{ijsr}{\sum }\left[(a_{ijs}-\gamma_{s}p_{ijs})+\delta (\sigma_{is},\sigma_{js})+\delta (i,j).\;\omega_{jrs}\right]\delta (\sigma_{is},\sigma_{jr})$$

where nodes *i* and *j* are assigned to modules σ_*is*_ and σ_*js*_ in window *s*, respectively. *A*_*ijs*_ represents the weight of the edge between these two nodes in window *s*; γ is the structural resolution parameter; ω is called the “inter-layer coupling strength,” and links the same node *j* within the network evaluated at two different times (“layers”): therefore, ω_*jrs*_ links the node *j* at times *r* and *s*, that is, in layer *r* and in layer *s*. Here, we chose γ = ω_*jrs*_ = 1; *p*_*ijs*_ represents the expected number of links according to a null model. As a reminder, the Kronecker δ-function is such that δ(*x*, *y*) is 1 if *x* = *y*, and 0 otherwise.

The multilayer network modularity was computed 100 times since Q may vary from run to run (degeneracy). This step is mandatory precisely because the degeneracy intrinsic to the community detection algorithm results in different networks structures depending on the run. Classically, this issue is dealt with using the so-called “co-classification matrix” or “consensus matrix,” whose elements indicate the ratio of each region to be in the same module with the other regions among these 100 partitions. Only elements in the consensus matrix higher than an appropriate random null model were considered. The randomized networks (generated with the null model) have the same strength distribution as the true brain networks. To quantify the dynamics of brain networks, we used the flexibility metric proposed in Bassett et al. (2011). The flexibility of a brain region is defined as the number of times that a brain region changed modular assignment throughout the session, normalized by the total number of changes that were possible.

### Statistical Analysis

We assessed the correlation between the two conditions (resilience score vs. network were assessed using the Pearson correlation). We applied a FDR correction for multiple comparisons across regions (Benjamini & Hochberg, 1995).

### Software

The functional connectivity, network measures, and network visualization were performed using BCT (Rubinov & Sporns, 2010), EEGNET (Hassan et al., 2015), and BrainNet viewer (Xia et al., 2013), respectively. The Network Community Toolbox (http://commdetect.weebly.com/) was used to compute the consensus matrices as well as the values provided by the flexibility metrics.

## AUTHOR CONTRIBUTIONS

Véronique Paban: Conceptualization; Data curation; Funding acquisition; Methodology; Project administration; Resources; Writing – original draft. Julien Modolo: Formal analysis; Investigation; Visualization; Writing – review & editing. Ahmad Mheich: Data curation; Formal analysis; Software; Visualization. Mahmoud Hassan: Data curation; Formal analysis; Methodology; Software; Visualization; Writing – review & editing.

## TECHNICAL TERMS

Resilience: One of the main personality traits, describing the capability of individuals to cope with stress and life changes.
EEG: Electroencephalography is a neuroimaging technique consisting of recording with scalp electrodes the small currents induced by neuronal activity at the cortex level.
Source-space networks: Networks formed by interacting anatomical brain regions, as opposed to sensor-level networks.
Flexibility: Metric from graph theory quantifying how often one node of a network changes its affiliation with a module over time.
Rich club: Subset of cortical regions that are extensively connected with other brain regions.
Static network analysis: Analysis of the brain network involving some form of averaging over time.
Dynamic network analysis: Analysis of the brain network made using time-dependent estimates of the network, as opposed to static network analysis.

## References

[bib1] Bassett D. S., & Sporns O. (2017). Network neuroscience. Nature Neuroscience, 20, 353. 28230844 10.1038/nn.4502 PMC5485642

[bib2] Bassett D. S., Wymbs N. F., Porter M. A., Mucha P. J., Carlson J. M., & Grafton S. T. (2011). Dynamic reconfiguration of human brain networks during learning. Proceedings of the National Academy of Sciences of the United States of America, 108(18), 7641–7646. 21502525 10.1073/pnas.1018985108 PMC3088578

[bib3] Benjamini Y., & Hochberg Y. (1995). Controlling the false discovery rate: A practical and powerful approach to multiple testing. Journal of the Royal Statistical Society B, 57(1), 289–300.

[bib4] Betzel R. F., Fukushima M., He Y., Zuo X.-N., & Sporns O. (2016). Dynamic fluctuations coincide with periods of high and low modularity in resting-state functional brain networks. NeuroImage, 127, 287–297. 26687667 10.1016/j.neuroimage.2015.12.001 PMC4755785

[bib5] Bonanno G. A. (2004). Loss, trauma, and human resilience: Have we underestimated the human capacity to thrive after extremely aversive events? American Psychologist, 59, 20. 14736317 10.1037/0003-066X.59.1.20

[bib6] Bonnefond M., Kastner S., & Jensen O. J. E. (2017). Communication between brain areas based on nested oscillations. ENEURO, 0153–0116.2017. 10.1523/ENEURO.0153-16.2017 PMC5367085 28374013

[bib7] Brookes M. J., Woolrich M. W., & Price D. (2014). An introduction to MEG connectivity measurements. In Magnetoencephalography (pp. 321–358). Berlin: Springer.

[bib8] Bush G., Luu P., & Posner M. I. (2000). Cognitive and emotional influences in anterior cingulate cortex. Trends in Cognitive Sciences, 4, 215–222. 10827444 10.1016/s1364-6613(00)01483-2

[bib9] Campbell-Sills L., & Stein M. B. (2007). Psychometric analysis and refinement of the Connor-Davidson Resilience Scale (CD-RISC): Validation of a 10-item measure of resilience. Journal of Traumatic Stress, 20, 1019–1028. 18157881 10.1002/jts.20271

[bib10] Colclough G., Brookes M., Smith S., & Woolrich M. (2015). A symmetric multivariate leakage correction for MEG connectomes. NeuroImage, 117, 439–448. 25862259 10.1016/j.neuroimage.2015.03.071 PMC4528074

[bib11] Connor K. M., & Davidson J. R. (2003). Development of a new resilience scale: The Connor-Davidson Resilience Scale (CD-RISC). Depression and Anxiety, 18, 76–82. 12964174 10.1002/da.10113

[bib12] Davydov D. M., Stewart R., Ritchie K., & Chaudieu I. (2010). Resilience and mental health. Clinical Psychology Review, 30, 479–495. 20395025 10.1016/j.cpr.2010.03.003

[bib13] Desikan R. S., Ségonne F., Fischl B., Quinn B. T., Dickerson B. C., Blacker D., Buckner R. L., Dale A. M., Maguire R. P., Hyman B. T., Albert M. S., & Killiany R. J. (2006). An automated labeling system for subdividing the human cerebral cortex on MRI scans into gyral based regions of interest. NeuroImage, 31, 968–980. 16530430 10.1016/j.neuroimage.2006.01.021

[bib14] Douw L., Nieboer D., Stam C. J., Tewarie P., & Hillebrand A. (2018). Consistency of magnetoencephalographic functional connectivity and network reconstruction using a template versus native MRI for co-registration. Human Brain Mapping, 39, 104–119. 28990264 10.1002/hbm.23827 PMC5725722

[bib15] Dubois J., Galdi P., Han Y., Paul L. K., & Adolphs R. (2018). Resting-state functional brain connectivity best predicts the personality dimension of openness to experience. Personality Neuroscience, 1:e6. 10.1017/pen.2018.8 PMC6138449 30225394

[bib16] Egner T., Monti J. M., Trittschuh E. H., Wieneke C. A., Hirsch J., & Mesulam M.-M. (2008). Neural integration of top-down spatial and feature-based information in visual search. Journal of Neuroscience, 28, 6141–6151. 18550756 10.1523/JNEUROSCI.1262-08.2008 PMC6670545

[bib17] Fletcher D., & Sarkar M. (2013). Psychological resilience: A review and critique of definitions, concepts, and theory. European Psychologist, 18, 12.

[bib18] Gramfort A., Papadopoulo T., Olivi E., & Clerc M. (2010). OpenMEEG: Opensource software for quasistatic bioelectromagnetics. Biomedical Engineering Online, 9, 45. 20819204 10.1186/1475-925X-9-45 PMC2949879

[bib19] Guihard G., Deumier L., Alliot-Licht B., Bouton-Kelly L., Michaut C., & Quilliot F. (2018). Psychometric validation of the French version of the Connor-Davidson Resilience Scale. L’Encéphale, 44, 40–45. 10.1016/j.encep.2017.06.002 28870690

[bib20] Hassan M., Dufor O., Merlet I., Berrou C., & Wendling F. (2014). EEG source connectivity analysis: From dense array recordings to brain networks. PloS One, 9, e105041. 25115932 10.1371/journal.pone.0105041 PMC4130623

[bib21] Hassan M., Merlet I., Mheich A., Kabbara A., Biraben A., Nica A., & Wendling F. (2017). Identification of interictal epileptic networks from Dense-EEG. Brain Topography, 30, 60–76. 27549639 10.1007/s10548-016-0517-z

[bib22] Hassan M., Shamas M., Khalil M., El Falou W., & Wendling F. (2015). EEGNET: An open source tool for analyzing and visualizing M/EEG connectome. PloS One, 10, e0138297. 26379232 10.1371/journal.pone.0138297 PMC4574940

[bib23] Hassan M., & Wendling F. (2018). Electroencephalography source connectivity: Aiming for high resolution of brain networks in time and space. IEEE Signal Processing Magazine, 35, 81–96.

[bib24] Hong L. E., Summerfelt A., Mitchell B. D., McMahon R. P., Wonodi I., Buchanan R. W., & Thaker G. K. (2008). Sensory gating endophenotype based on its neural oscillatory pattern and heritability estimate. Archives of General Psychiatry, 65, 1008–1016. 18762587 10.1001/archpsyc.65.9.1008 PMC2774756

[bib25] Kabbara A., Eid H., El Falou W., Khalil M., Wendling F., & Hassan M. (2018). Reduced integration and improved segregation of functional brain networks in Alzheimer’s disease. Journal of Neural Engineering, 15, 026023. 29451125 10.1088/1741-2552/aaaa76

[bib26] Kabbara A., Falou W. E., Khalil M., Wendling F., & Hassan M. (2017). The dynamic functional core network of the human brain at rest. Scientific Reports, 7, 2936. 28592794 10.1038/s41598-017-03420-6 PMC5462789

[bib27] Kim M., & Maguire E. A. (2018). Thalamus, subiculum and retrosplenial cortex encode 3D head direction information in volumetric space. bioRxiv, 335976.

[bib28] Kong F., Wang X., Hu S., & Liu J. (2015). Neural correlates of psychological resilience and their relation to life satisfaction in a sample of healthy young adults. NeuroImage, 123, 165–172. 26279212 10.1016/j.neuroimage.2015.08.020

[bib29] Lachaux J. P., Rodriguez E., Martinerie J., & Varela F. J. (1999). Measuring phase synchrony in brain signals. Human Brain Mapping, 8, 194–208. 10619414 10.1002/(SICI)1097-0193(1999)8:4<194::AID-HBM4>3.0.CO;2-C PMC6873296

[bib30] Lachaux J.-P., Rodriguez E., Le Van Quyen M., Lutz A., Martinerie J., & Varela F. J. (2000). Studying single-trials of phase synchronous activity in the brain. International Journal of Bifurcation and Chaos, 10, 2429–2439.

[bib31] Lai M., Demuru M., Hillebrand A., & Fraschini M. (2018). A comparison between scalp- and source-reconstructed EEG networks. Scientific Reports, 8, 12269. 30115955 10.1038/s41598-018-30869-w PMC6095906

[bib32] Lisman J. E., & Jensen O. J. N. (2013). The theta-gamma neural code. Neuron, 77(6), 1002–1016. 23522038 10.1016/j.neuron.2013.03.007 PMC3648857

[bib33] Liu J. J., Reed M., & Girard T. A. (2017). Advancing resilience: An integrative, multi-system model of resilience. Personality and Individual Differences, 111, 111–118.

[bib34] Luthar S. S. (2003). Resilience and Vulnerability: Adaptation in the Context of Childhood Adversities. Cambridge, UK: Cambridge University Press.

[bib35] Markett S., Montag C., & Reuter M. (2018). Network neuroscience and personality. Personality Neuroscience, 1. 10.1017/pen.2018.12 PMC7219685 32435733

[bib36] Mheich A., Hassan M., Khalil M., Gripon V., Dufor O., & Wendling F. (2018). SimiNet: A novel method for quantifying brain network similarity. IEEE Transactions on Pattern Analysis and Machine Intelligence, 40, 2238–2249. 28910755 10.1109/TPAMI.2017.2750160

[bib37] Mucha P. J., Richardson T., Macon K., Porter M. A., & Onnela J.-P. (2010). Community structure in time-dependent, multiscale, and multiplex networks. Science, 328, 876–878. 20466926 10.1126/science.1184819

[bib38] Paban V., Deshayes C., Ferrer M.-H., Weill A., & Alescio-Lautier B. (2018). Resting brain functional networks and trait coping. Brain Connectivity, 8(8), 475–486. 30221547 10.1089/brain.2018.0613

[bib39] Paban V., Modolo J., Mheich A., & Hassan M. (2019). Supporting Information for “Psychological resilience correlates with EEG source-space brain network flexibility.” Network Neuroscience, 3(2), 539–550. 10.1162/netn_a_00079 PMC6444909 30984906

[bib40] Reynaud E., Guedj E., Souville M., Trousselard M., Zendjidjian X., El Khoury-Malhame M., … Canini F. (2013). Relationship between emotional experience and resilience: An fMRI study in fire-fighters. Neuropsychologia, 51, 845–849. 23369802 10.1016/j.neuropsychologia.2013.01.007

[bib41] Rizkallah J., Benquet P., Kabbara A., Dufor O., Wendling F., & Hassan M. (2018). Dynamic reshaping of functional brain networks during visual object recognition. Journal of Neural Engineering, 15(5). 10.1088/1741-2552/aad7b1 30070974

[bib42] Rubinov M., & Sporns O. (2010). Complex network measures of brain connectivity: Uses and interpretations. NeuroImage, 52, 1059–1069. 19819337 10.1016/j.neuroimage.2009.10.003

[bib43] Rutter M. (2006). Implications of resilience concepts for scientific understanding. Annals of the New York Academy of Sciences, 1094, 1–12. 10.1196/annals.1376.002 17347337

[bib44] Scali J., Gandubert C., Ritchie K., Soulier M., Ancelin M.-L., & Chaudieu I. (2012). Measuring resilience in adult women using the 10-items Connor-Davidson Resilience Scale (CD-RISC). Role of trauma exposure and anxiety disorders. PloS One, 7, e39879. 22768152 10.1371/journal.pone.0039879 PMC3387225

[bib45] Schoffelen J. M., & Gross J. (2009). Source connectivity analysis with MEG and EEG. Human Brain Mapping, 30, 1857–1865. 19235884 10.1002/hbm.20745 PMC6870611

[bib46] Schuck N. W., Wilson R., & Niv Y. (2018). A state representation for reinforcement learning and decision-making in the orbitofrontal cortex. In Goal-Directed Decision Making (pp. 259–278). Amsterdam: Elsevier.

[bib47] Sestieri C., Shulman G. L., & Corbetta M. (2017). The contribution of the human posterior parietal cortex to episodic memory. Nature Reviews Neuroscience, 18, 183. 28209980 10.1038/nrn.2017.6 PMC5682023

[bib48] Sporns O., & Betzel R. F. (2016). Modular brain networks. Annual Review of Psychology, 67, 613–640. 10.1146/annurev-psych-122414-033634 PMC4782188 26393868

[bib49] Tadel F., Baillet S., Mosher J. C., Pantazis D., & Leahy R. M. (2011). Brainstorm: A user-friendly application for MEG/EEG analysis. Computational Intelligence and Neuroscience, 2011, 8. 10.1155/2011/879716 PMC3090754 21584256

[bib50] Tompson S., Falk E. B., Vettel J. M., & Bassett D. S. (2018). Network approaches to understand individual differences in brain connectivity: Opportunities for personality neuroscience. Personality Neuroscience, 1:e5. 10.1017/pen.2018.4 PMC6133307 30221246

[bib51] Van Den Heuvel M. P., & Sporns O. (2011). Rich-club organization of the human connectome. Journal of Neuroscience, 31, 15775–15786. 22049421 10.1523/JNEUROSCI.3539-11.2011 PMC6623027

[bib52] van der Werff S. J., van den Berg S. M., Pannekoek J. N., Elzinga B. M., & Van Der Wee N. J. (2013). Neuroimaging resilience to stress: A review. Frontiers in Behavioral Neuroscience, 7, 39. 23675330 10.3389/fnbeh.2013.00039 PMC3646289

[bib53] Vidaurre D., Hunt L. T., Quinn A. J., Hunt B. A., Brookes M. J., Nobre A. C., & Woolrich M. W. (2018). Spontaneous cortical activity transiently organises into frequency specific phase-coupling networks. Nature Communications, 9, 2987. 10.1038/s41467-018-05316-z PMC6065434 30061566

[bib54] Waugh C. E., & Koster E. H. (2015). A resilience framework for promoting stable remission from depression. Clinical Psychology Review, 41, 49–60. 24930712 10.1016/j.cpr.2014.05.004

[bib55] Xia M., Wang J., & He Y. (2013). BrainNet Viewer: A network visualization tool for human brain connectomics. PloS One, 8, e68910. 23861951 10.1371/journal.pone.0068910 PMC3701683

