# Supplementary material for: Psychological resilience correlates with EEG source-space brain network flexibility
Source: Netw Neurosci. 2019 Mar 1;3(2):539–50. doi: 10.1162/netn_a_00079 (PMC6444909; doi:10.1162/netn_a_00079)
Supplement: Supplementary file 1 [file netn-03-539-s001.pdf]

Paban, V., Modolo, J., Mheich, A., & Hassan, M. (2019). Supporting information for "Psychological resilience correlates with EEG source-space brain network flexibility." *Network Neuroscience*, *3*(2), 539–550. https://doi.org/10.1162/netn_a_00079

Supplementary materials

Psychological resilience correlates with EEG source-space brain network flexibility

Veronique Paban[1], Julien Modolo[2], Ahmad Mheich[2], Mahmoud Hassan[2]

[1]Aix Marseille Univ, CNRS, LNSC, Marseille France

[2] Univ Rennes, INSERM, LTSI - U1099, F-35000 Rennes, France

Corresponding author:

Julien Modolo – julien.modolo@inserm.fr - Laboratoire Traitement du Signal et de l'Image (LTSI), Bâtiment 22 Campus de Beaulieu, 35042 Rennes Cedex, France

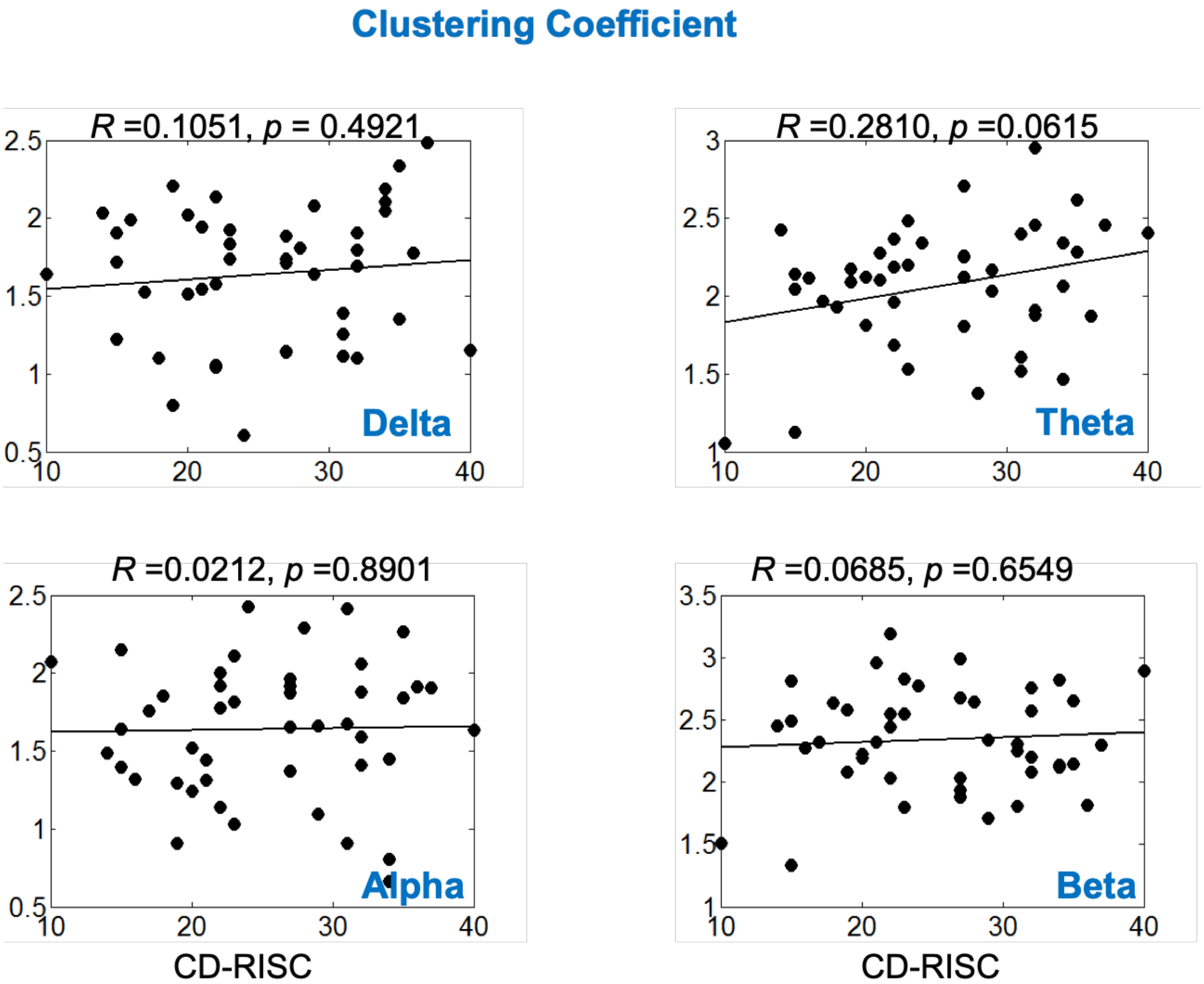

Figure S1. Correlation between the Resilience score and the global clustering coefficients at the different frequency bands.

Betweenness Centrality

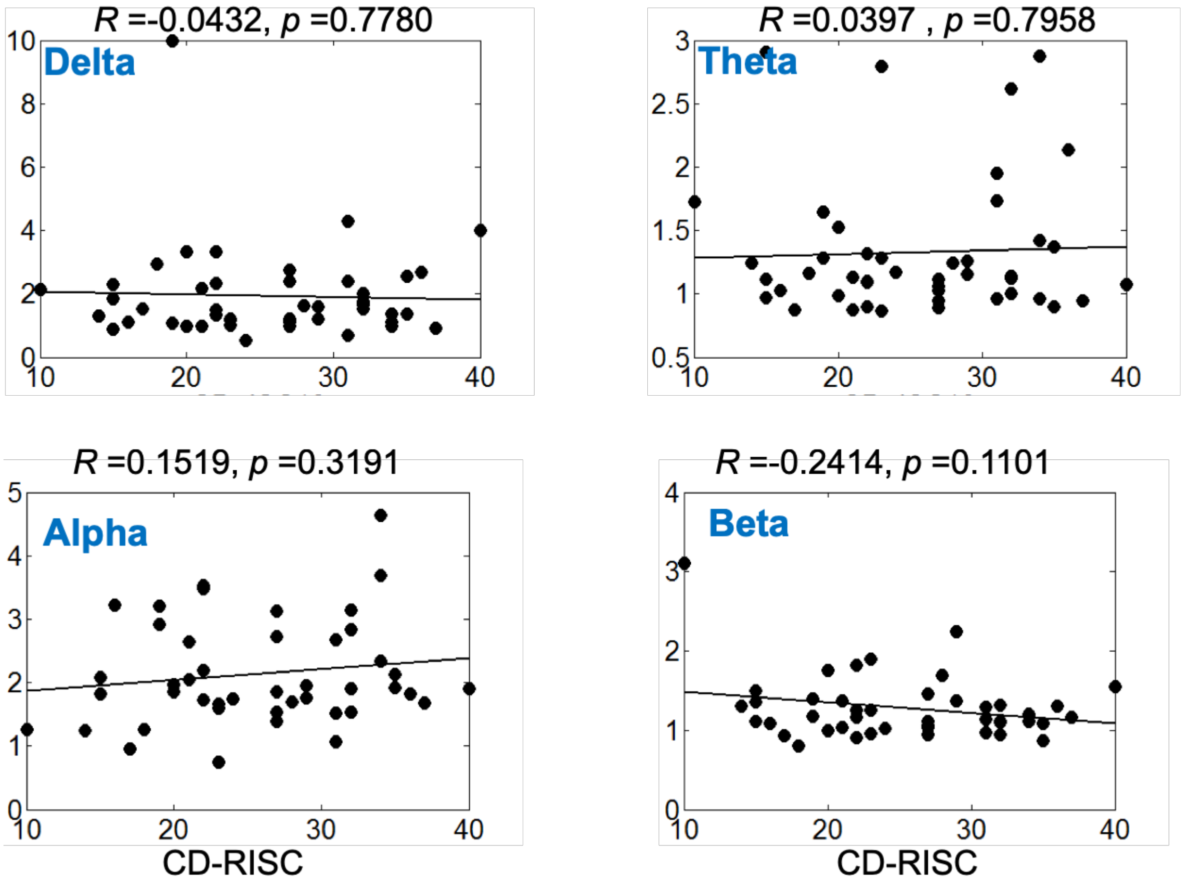

Figure S2. Correlation between the Resilience score and the global betweenness centrality at the different frequency bands.
